# A comparison of UK primary care data with other national data sources for monitoring the prevalence of smoking during pregnancy

**DOI:** 10.1093/pubmed/fdu060

**Published:** 2014-10-21

**Authors:** Nafeesa N. Dhalwani, Laila J. Tata, Tim Coleman, Linda Fiaschi, Lisa Szatkowski

**Affiliations:** 1 Division of Epidemiology and Public Health, University of Nottingham, Clinical Sciences Building, Nottingham City Hospital, Hucknall Road, Nottingham NG5 1PB, UK; 2 Division of Primary Care, University of Nottingham, Queen's Medical Centre, Nottingham NG7 2UH, UK

**Keywords:** primary care, pregnancy and childbirth disorders, smoking

## Abstract

**Background:**

We aimed to assess the potential usefulness of primary care data in the UK for estimating smoking prevalence in pregnancy by comparing the primary care data estimates with those obtained from other data sources.

**Methods:**

In The Health Improvement Network (THIN) primary care database, we identified pregnant smokers using smoking information recorded during pregnancy. Where this information was missing, we used smoking information recorded prior to pregnancy. We compared annual smoking prevalence from 2000 to 2012 in THIN with measures from the Infant Feeding Survey (IFS), Smoking At Time of Delivery (SATOD), Child Health Systems Programme (CHSP) and Scottish Morbidity Record (SMR).

**Results:**

Smoking estimates from THIN data converged with estimates from other sources after 2004, though still do not agree completely. For example, in 2012 smoking prevalence at booking was 11.6% in THIN using data recorded only during pregnancy, compared with 19.6% in SMR data. However, the use of smoking data recorded up to 27 months before conception increased the THIN prevalence to 20.3%, improving the comparability.

**Conclusions:**

Under-recording of smoking status during pregnancy results in unreliable prevalence estimates from primary care data and needs improvement. However, in the absence of gestational smoking data, the inclusion of pre-conception smoking records may increase the utility of primary care data. One strategy to improve gestational smoking status recording in primary care could be the inclusion of pregnancy in the Quality and Outcome’s Framework as a condition for which smoking status and smoking cessation advice must be recorded electronically in patient records.

## Introduction

Smoking in pregnancy is an important preventable cause of poor health outcomes for women and their babies.^1,2^ In March 2011, the UK Government white paper entitled ‘Healthy lives healthy people: A tobacco control plan for England’ set out a national goal to reduce the prevalence of smoking throughout pregnancy to 11% or less by 2015.^3^ It is therefore crucial to collect data on maternal smoking to monitor progress towards this national goal. The UK currently has four data sources that provide population-level estimates of smoking during pregnancy. Each measures smoking differently and has its strengths and limitations The Infant Feeding Survey (IFS) measures smoking at delivery, retrospectively, at 6–8 weeks postpartum in the UK.^4–6^ The smoking at the time of delivery (SATOD) data measure smoking behaviour at the time of delivery,^7^ whereas the Child Health Systems Programme (CHSP) Pre-School Component measures maternal smoking around delivery usually within 10 days postpartum.^8^ In comparison, data from the Scottish Morbidity Records (SMR) measure smoking at the time of first antenatal appointment. Electronic primary care records contain routinely collected information on medical diagnoses, prescriptions and other data such as patients' smoking status,^9^ and thus could potentially provide comprehensive and timely population-level data on smoking prevalence during pregnancy. In April 2004, a contract for UK general practitioners (GPs) (family physicians) was implemented; this introduced pay-for-performance targets known as the Quality and Outcomes Framework (QOF)^10^ according to which the recording of smoking status and recorded delivery of smoking cessation advice can generate revenue of up to £10 000 per year per practice.^11,12^ Consequently, the recording of smoking status in primary care data has improved such that, outside of pregnancy, UK primary care data are a valid source of data to monitor smoking prevalence at a population level both nationally and regionally.^13,14^ However, the potential use of these data for generating estimates of smoking during pregnancy at a population level is yet to be studied. In an earlier study, we found that the recording of smoking status during pregnancy is relatively incomplete; in 2009, only 43% of women had a record for smoking status during pregnancy.^15^ However, in this previous work, we found that the utility of incomplete individual-level smoking status data could be improved by making various assumptions which reflected data recording practices encouraged by the QOF.^15^ Consequently, in this paper, we test similar assumptions to assess the potential usefulness of primary care data for estimating the population smoking prevalence in pregnancy by comparing estimates from primary care data with those obtained from other available data sources.

## Methods

### Data source and study population

The Health Improvement Network (THIN) is an electronic primary care database containing anonymized patient records from general practices across the UK. It is representative of the UK population in terms of patient demographics and the prevalence of common illnesses.^16^ The version of THIN used for this study contained data from 570 practices, covering ∼6% of the UK population.^9^ Our study population included all women of reproductive age (defined as 15–49^17^) in THIN with pregnancies ending in live births or stillbirths from 2000 to 2012. Pregnancies ending in miscarriage were not included in the study population as many of these occur early in pregnancy when women may not know they are pregnant. Therefore, they may not be reported to the doctor, or if they are reported, the first consultation indicating the pregnancy may be for reporting the miscarriage, when ascertainment of smoking status would only be retrospective. For women with more than one pregnancy during the study time, one pregnancy was chosen at random for analysis to prevent any clustering effects.

### Comparing the prevalence of smoking in pregnancy in THIN with other data sources

For each woman, we extracted all records of smoking status recorded in THIN using Read codes^18^ before and during pregnancy and up to 10 days after delivery (e.g. 137R.00—Current smoker). Where a Read code did not clearly indicate current smoking (e.g. 137X.00—Cigarette consumption), we assessed whether smoking status could be derived from any additional information recorded, such as the number of cigarettes smoked, or the presence of prescriptions for smoking cessation medications. If no additional information was found, the recording was labelled as unknown smoking status. Code lists are available from the authors on request.

Using a previously validated algorithm,^13^ we used the extracted Read codes to determine each woman's smoking status during their pregnancy. The annual prevalence of smoking during pregnancy as recorded in THIN (as a proportion of all births in that year) was then compared against the prevalence measures from the IFS, SATOD, SMR and CHSP. A detailed description of each of these data sources is provided in Table 1.

**Table 1:** Summary of available data sources to measure smoking during pregnancy in the UK

*Data source*	*Data collection interval*	*Country*	*Sampling frame and method*	*Sample size ^a^ (% of national births)*	*Data collection method*	*Time at which data on smoking in pregnancy are collected*	*Definition of smoking*	*Strengths*	*Limitations*
Infant Feeding Survey^4–6^	Every 5 years	UK (England, Scotland, Wales, Northern Ireland)	Random sample of live births in England and Scotland and all births in Wales and Northern Ireland in study period	22 400 (2.7% of all births in the UK)^19–23^	Postal survey administered by the National Health Service Information Centre	6–8 weeks after birth	Several self-reported measures available: smoking prior to pregnancy; ever smoking during pregnancy; quitting on confirmation of pregnancy; quit/cut down attempts during pregnancy; smoking at delivery.	Smoking estimates for overall UK and each constituent country Smoking status presented by sociodemographic factors Measures smoking cessation during pregnancy	Data only collected at 5 years intervals Retrospective reporting of smoking status Low response rates (∼52%) Results published at least a year after survey completion
Smoking Status at Time of Delivery (SATOD)^7^	Collected continually and reported quarterly	England	Aims to capture all live births and stillbirths	359 763 (52.1% of all births in England)^19,22^	Midwife survey (in hospital maternity units)	At delivery	Self-reported smoking status at delivery	Data collected and reported at a local level	Limited to England Data collected postnatally No assessment of smoking by sociodemographic factors
Smoking Data collected as part of the Scottish Morbidity Record (SMR)^8^	Collected continually and reported by financial year	Scotland	All pregnant women attending an antenatal booking appointment (pregnancies may end in live birth or stillbirth)	57 398 (100% of all maternities in Scotland)^20,23^	Midwife survey (in hospital or community)	First antenatal booking appointment (usually between 8–12 weeks gestation)	Self-reported smoking status at the time of booking	Provides measures of never/ex smoking along with current smoking Provides annual rates by age and socio-economic status	Limited to Scotland Does not give estimates for the whole duration of pregnancy
Pre-school component of the Child Health Systems Programme (CHSP)^8^	Collected continually and reported by financial year	Scotland	Aims to capture all live births	51 746 (92% of all live births in Scotland) ^20,23^	Survey administered by public health nurse or health visitor	Approximately 10 days after birth	Self-reported smoking status at the time of survey ∼10 days after delivery	Provides data on smokers and non-smokers by age and socio-economic status	Limited to Scotland Data collected postnatally only Does not specifically ask about smoking during pregnancy

^a^Sample sizes for each wave vary therefore sample sizes for 2010 described in the table for reference.

Each comparison used a slightly different population of women from THIN and assessed smoking status at a different point in time in pregnancy to reflect the nature of the data collection in the source being compared (see Table 2). Estimates of smoking prevalence from the IFS were derived from the ‘raw’ data sets of individual women's survey responses, available from the UK Data Service.^24^ The IFS only asked about smoking status retrospectively, so women were classified as smoking at delivery if they reported that they tried to give up smoking during pregnancy but started again before delivery, if they tried to cut down on the amount smoked during pregnancy, or if they did not try to cut down during pregnancy. Estimates of the prevalence of smoking from SATOD, SMR and CHSP data were obtained from published reports.

**Table 2:** THIN comparisons with the currently available data in the UK

*Survey*	*Time at which survey assesses smoking prevalence*	*Years compared with THIN*	*THIN population used for comparison*	*Timing of records considered to define smoking status in THIN*
Infant Feeding Survey (IFS)	At delivery	2000, 2005, 2010	Data from all UK practices (*n* = 570)	Last smoking status recording between conception and delivery
Smoking Status at Time of Delivery (SATOD)	At delivery	2006–2012	Data from English practices (*n* = 420)	Last smoking status recording between conception and delivery
Scottish Morbidity Record (SMR)	At booking (8–12 weeks gestation)	2000–2012	Data from Scottish practices (*n* = 85)	First smoking status recording between conception and delivery
Child Health Systems Programme (CHSP)	10 days after delivery	2001–2012	Data from Scottish practices (*n* = 85)	Last smoking status recording between conception and 10 days after delivery

### Imputing smoking status where women had no record during the gestational period

Initially, we used only records of smoking status documented in the primary care record after the date of conception to determine smoking status during pregnancy. However, if a woman's smoking status was not recorded during gestation, we used pre-conception records of smoking status to identify women who might have smoked during pregnancy. Based on the QOF rules for the recording of smoking status in the general population, which from April 2004 to March 2006 required the smoking status of patients aged 15 or over to be recorded at least once in primary care records, and since April 2006 have required records to be updated every 27 months, we used two cut-off points for including information from pre-conception records.^25^ Firstly, we used a cut-off of 27 months before conception and recoded women as smokers if their last smoking record in the 27 months before conception indicated smoking. Finally, if a woman did not have her smoking status recorded either during pregnancy or in the 27 months before conception, we included any smoking information recorded in their primary care data since they registered with their practice.

All analyses were conducted in Stata 12.0 (StataCorp LP, College Station, TX, USA). Ethical approval was obtained from the THIN Scientific Review Committee (Reference number 11-047).

## Results

### Population of pregnancies and smoking in THIN

We identified 310 043 women with one or more pregnancies ending in a live birth or stillbirth from 2000 to 2012; 246 730 of these women were registered with a GP in England and 34 442 were in Scotland. The mean age at conception was 29.5 years (standard deviation 5.9 years). Only 30% of women had their smoking status recorded at least once during pregnancy and of these women 75% only had a single record.

### Comparison with IFS data

**Fig. 1:**
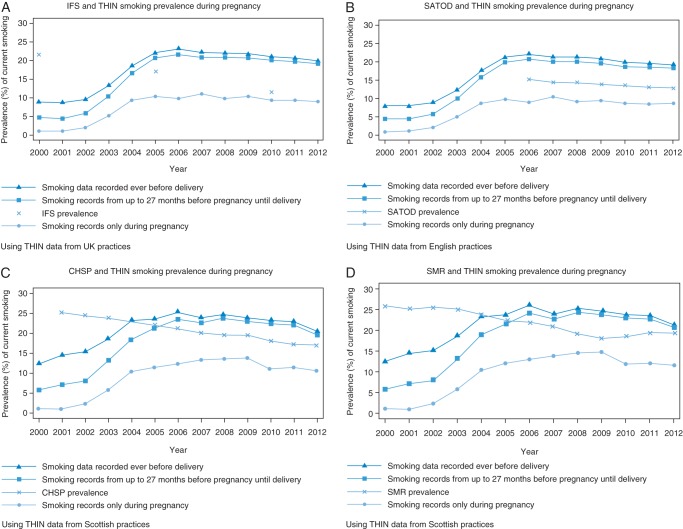
Comparison of smoking prevalence from currently available data sources and THIN.

Figure 1a shows the prevalence of smoking at the time of delivery in women in THIN compared with the prevalence measures in the IFS. Annual trends could not be compared as there were only three data points available. In 2000, none of the three prevalence estimates using THIN data were comparable with the IFS estimates. In 2005, smoking prevalence including data recorded up to 27 months before conception from THIN was slightly higher than the IFS estimate (17.0 versus 20.6%, respectively). In 2010, the IFS prevalence of smoking at the time of delivery decreased further to 11.6%, while the THIN prevalence using data recorded up to 27 months before conception remained similar (19.9%). In comparison, the IFS prevalence for 2010 was ∼3 percentage points higher than the THIN prevalence using only smoking data recorded during pregnancy (11.6% in the IFS compared with 9.3% in THIN).

### Comparison with SATOD data

When using smoking data recorded any time before delivery, the prevalence of smoking during pregnancy recorded in THIN was ∼7 percentage points higher than the SATOD estimates from 2006 to 2012. In comparison, the THIN prevalence considering data recorded up to 27 months before conception was ∼4–5 percentage points higher over the 6 years of available data, while the THIN prevalence considering only records of smoking recorded during the gestational period was 4–5 percentage points lower than the SATOD estimates (Fig. 1b).

### Comparison with CHSP data

Using only records of smoking status entered during the gestational period, the THIN prevalence of maternal smoking was low until 2004 (e.g. 44% of the CHSP prevalence of 23.1% in 2004) (Fig. 1c). It was 10.5% in 2012, ∼7 percentage points lower than the corresponding CHSP prevalence of 17.1%. Using smoking information recorded in the 27 months before pregnancy, the prevalence in CHSP and THIN converged in 2005. After this, the THIN estimates were slightly higher than the CHSP estimates, such that in 2012 the THIN prevalence using data recorded up to 27 months before pregnancy was 19.9% compared with the CHSP prevalence of 17.1%. The prevalence estimates using data recorded ever before delivery were only slightly higher than the estimates using data recorded up to 27 months before conception.

### Comparison with SMR data

Using smoking status data recorded during the gestational period, the THIN prevalence was much lower than the SMR prevalence until 2004 (THIN prevalence = 10.6% compared with SMR prevalence of 23.8% in 2004, as shown in Fig. 1d). Prevalence in THIN was 11.6% in 2012 but was still 40% lower than the corresponding SMR prevalence of 19.6%. When including smoking information recorded up to 27 months before conception, the two lines converged between 2004 and 2005; in 2012, smoking prevalence in THIN was 20.3% using data recorded up to 27 months before conception and smoking prevalence using data recorded any time before pregnancy was 21.3% compared with the SMR prevalence of 19.3%.

## Discussion

### Main findings

We found that, with current levels of completeness of smoking data in primary care records, it is not possible to produce population level estimates for smoking prevalence during pregnancy that are directly comparable with those derived from existing surveys. The convergence between THIN estimates and estimates from other data sources has, however, improved over time especially following the introduction of the QOF. Data from the IFS show good agreement with smoking at delivery in women in 2010 as recorded in THIN based on smoking status records entered in the electronic medical record during pregnancy. THIN data, using smoking data recorded up to 27 months before conception, show good agreement with SMR estimates in the final year of the study period.

### What is already known on the topic

To date, there are no studies assessing the validity of primary care data for quantifying the prevalence of smoking during pregnancy. A study comparing smoking prevalence recorded in THIN to smoking prevalence in the general population [measured by the General Lifestyle Survey (GLF)] found a good agreement between THIN and the GLF after 2008 and concluded that primary care data may provide an alternate means of monitoring national smoking prevalence.^18^ Despite the smaller sample sizes at regional level, primary care data have also been shown to be a good means of monitoring regional smoking prevalence in the general population.^19^

If primary care data were valid to monitor smoking prevalence during pregnancy, there would be several advantages of using these data to do so. All women in the UK must be registered with a GP in pregnancy to receive free antenatal care, so their records will be available in GP research databases. THIN data are routinely collected, have a lag of only 3–8 months before clinical data become available to researchers, and have the statistical power to provide estimates for the whole UK as well as constituent countries.^18^

### What this study adds

The prevalence estimates of smoking during pregnancy from primary care do not accurately converge with other data sources because, at least in part, smoking status recording during pregnancy in primary care is incomplete.^20^ If a woman's status did not change after she became pregnant (e.g. a non-smoker before pregnancy remained a non-smoker during pregnancy, or a smoker continued to smoke), GPs might be less likely to re-enter this information, which may account for the low completeness. Furthermore, in the UK, smoking status during pregnancy is primarily ascertained by midwives and recorded in women's handheld maternity records [mandatory paper records that women carry throughout pregnancy as part of the UK's National Health Service (NHS) antenatal care]. While the National Institute for Health and Clinical Excellence (NICE) recommends that midwives and others involved in the care of pregnant women assess and document women's smoking status in their maternity records,^26,27^ this information is not routinely entered into primary care records as the documentation in midwives' notes is not usually transcribed onto the electronic primary care records. This was clearly reflected in our previous study which found that from 2000 to 2009 smoking status was only recorded in primary care for 28% of pregnancies.^20^ In the current study, smoking status was only recorded for 30% of pregnancies.

Another possible explanation for the lower THIN prevalence could be that THIN over-represents general practices from more affluent areas of the UK. Since smoking prevalence is lower in women from more affluent groups, this may slightly under-estimate the smoking prevalence generated using THIN data and account for some of the differences between THIN prevalence estimates and other data sources.

While THIN estimates using only gestational smoking records do not approximate closely to annual prevalence from other data sources, THIN estimates using smoking data from up to 27 months pre-conception are comparable with the SMR data (smoking status recorded at booking) in 2012. GP data may be most useful to provide adequate data on smoking prevalence early in pregnancy, when most women see their GPs for initial care, compared with the time around delivery, when most women will be cared for essentially in secondary care facilities.

### Limitations

This is the first study to assess the potential of primary care data to provide population-level estimates of smoking during pregnancy and compare it with other current data sources in the UK. Fertility rates in THIN are comparable with national fertility rates^28^ and therefore our ascertainment of pregnancies is valid. However, like the other data sources under comparison, data on smoking status recorded in THIN are self-reported and women may not accurately report their smoking behaviour, particularly during pregnancy where there may be social stigma attached to smoking.^29^

A potential limitation of our study was the inclusion of pre-conception smoking records to predict smoking status during pregnancy, which may not be an accurate reflection of women's smoking status during pregnancy. Studies which have investigated smoking behaviour in early pregnancy indicate that many women attempt to quit when they find out they are pregnant or later during pregnancy,^30^ so it is unlikely that the inclusion of pre-conception records resulted in an under-estimation of smoking prevalence during pregnancy. It could however, lead to misclassification of some ex-smokers as current smokers, resulting in an over-estimation of the prevalence of current smoking during pregnancy in THIN. We believe that a substantial over-estimation is unlikely as ∼35–50% of pregnancies in the UK are unplanned,^31,32^ which means that only some women are likely to make positive behaviour changes such as quitting smoking before attempting to conceive. It may, however, hold true for some women who quit on confirmation of their pregnancy.

Another potential weakness of our study, and of primary care data itself, is that it is difficult to determine the timing of smoking status ascertainment in relation to progress through gestation; this makes direct comparison with other data sources, obtained at booking or delivery, difficult. Lastly, smoking status during pregnancy is a complex and variable behaviour and it may fluctuate throughout pregnancy.^33^ Therefore, single measures of smoking such as smoking at booking or smoking at delivery captured in SATOD, SMR and CHSP data are limited in their usefulness. Although they may give a snapshot of smoking behaviour at a certain time, they may not give a complete picture of smoking behaviour throughout pregnancy. IFS data assess smoking behaviour throughout pregnancy in more detail, albeit collected retrospectively. However, these data are collected on a quinquennial basis and thus may become out of date quickly. If smoking information was collected and recorded by GPs more frequently throughout pregnancy, then primary care data may prove to be very useful to assess the population-level burden of maternal smoking throughout pregnancy. However, as shown in this study, currently these data are not desirably complete.

### Conclusion

All existing data sources that measure smoking during pregnancy have their strengths and limitations. Primary care data have a great potential to measure smoking status during pregnancy at a population level, but this potential appears to be greatest for measuring smoking prevalence in early pregnancy around the time of booking appointments. Although recording of gestational smoking status in THIN is improving over time, it is not adequately complete to produce maternal smoking estimates at a population level with most women just having a single recording of smoking status throughout the course of pregnancy. Periodic recording of smoking status during pregnancy is important to monitor changes in smoking behaviour throughout pregnancy and to maintain and improve women's care before and after delivery. Although this information may be recorded and updated in handheld maternity notes, there is currently no centralized recording system and the information in these notes is lost after delivery. Better integration of recording systems in primary care and midwifery services is required to improve communication and relay of relevant medical and lifestyle information including smoking status. One strategy to improve this recording in primary care may be the inclusion of pregnancy in the QOF as a condition where smoking status and smoking cessation advice should be recorded in the electronic primary care records. This will not only increase opportunities for healthcare professionals to provide smoking cessation advice and interventions, but could also provide valuable data for the evaluation of the effectiveness of these interventions and monitoring progress towards meeting national prevalence targets.

## Funding

This work was supported by a University of Nottingham International Research Excellence Scholarship and the National Institute for Health Research (NIHR). This article presents independent research funded by the NIHR under its Programme Grants for Applied Research Programme (reference RP-PG 0109-10020). The views expressed in this article are those of the authors and not necessarily those of the NHS, the NIHR or the Department of Health. NND, TC and LS are members of the UK Centre for Tobacco and Alcohol Studies (UKCTAS), a UKCRC Public Health Research Centre of Excellence. Funding from the British Heart Foundation, Cancer Research UK, the Economic and Social Research Council, the Medical Research Council and the National Institute of Health Research, under the auspices of the UK Clinical Research Collaboration, is gratefully acknowledged. T.C. is also a member of the NIHR National School for Primary Care Research. The funders had no role in study design, data collection and analysis, decision to publish, or preparation of the manuscript.
